# Deep Learning Approaches for lncRNA-Mediated Mechanisms: A Comprehensive Review of Recent Developments

**DOI:** 10.3390/ijms241210299

**Published:** 2023-06-18

**Authors:** Yoojoong Kim, Minhyeok Lee

**Affiliations:** 1 School of Computer Science and Information Engineering, The Catholic University of Korea, Bucheon 14662, Republic of Korea; yoojoongkim@catholic.ac.kr; 2 School of Electrical and Electronics Engineering, Chung-Ang University, Seoul 06974, Republic of Korea

**Keywords:** deep learning, lncRNA, long non-coding RNA, gene transcription, protein regulation, machine learning

## Abstract

This review paper provides an extensive analysis of the rapidly evolving convergence of deep learning and long non-coding RNAs (lncRNAs). Considering the recent advancements in deep learning and the increasing recognition of lncRNAs as crucial components in various biological processes, this review aims to offer a comprehensive examination of these intertwined research areas. The remarkable progress in deep learning necessitates thoroughly exploring its latest applications in the study of lncRNAs. Therefore, this review provides insights into the growing significance of incorporating deep learning methodologies to unravel the intricate roles of lncRNAs. By scrutinizing the most recent research spanning from 2021 to 2023, this paper provides a comprehensive understanding of how deep learning techniques are employed in investigating lncRNAs, thereby contributing valuable insights to this rapidly evolving field. The review is aimed at researchers and practitioners looking to integrate deep learning advancements into their lncRNA studies.

## 1. Introduction

In the evolving landscape of machine learning, deep learning has revolutionized our understanding and application of technology, paving the way for breakthroughs and novel explorations in a plethora of fields [1,2,3,4]. It has spurred the advent of innovative methods, such as image generation [5,6,7] and natural language processing techniques [8,9,10,11], each contributing to expanding the horizons of deep learning applications. This emerging area of study has also found its relevance in molecular biology, particularly in understanding long non-coding RNAs (lncRNAs) [12,13,14].

Our focus in this review is on lncRNAs, which are a category of RNAs that lack protein-coding potential and consist of nucleotide sequences longer than 200 nucleotides, which are transcribed and processed mostly from intergenic regions, introns with or without some exons, or enhancer regions of the genome [12], yet have emerged as significant contributors to numerous biological processes. lncRNAs, with their intricate and diverse functions, have gained increasing attention from the scientific community as their aberrations have been implicated in various diseases. The fast-paced research dedicated to lncRNAs, in parallel with the rapid advancements in deep learning, necessitates a comprehensive overview of the intersection of these two vital areas of study.

As signal molecules, lncRNAs play a crucial role in the transcription of downstream genes, often exhibiting a high degree of context specificity [15,16,17]. Recent studies have shown lncRNAs to be highly organized in their transcription processes, responding and adapting to different environmental stimuli to influence particular signaling pathways [18,19,20]. This detailed orchestration of transcription by lncRNAs, often in association with specific proteins, such as transcription factors, underscores the integral role they play in transcriptional regulation.

In their capacity as decoy molecules, lncRNAs perform a variety of functions, including interference in various molecular pathways. These RNAs interact directly with specific protein molecules after their transcription, leading to a disruption in the normal functioning of these proteins [21,22]. This interaction with transcription regulators inhibits the transcription factors’ functionality, thus suppressing downstream gene transcription. Furthermore, lncRNAs can impede protein functionality, affecting their ability to regulate mRNA expression. Furthermore, lncRNAs have been found to play vital roles in tumor progression, participating in the regulation of gene expression at the epigenetic, transcriptional, and post-transcriptional levels [23,24]. Some lncRNAs influence gene expression by altering the chromatin structure, histone modification status, and DNA methylation status [25,26].

In the current technological landscape, deep learning has rapidly evolved to influence a range of scientific fields significantly. This remarkable evolution, combined with the emerging importance of lncRNAs, underscores the necessity to explore the confluence of these two spheres critically. Recognizing this need, we aim to deliver an exhaustive analysis of how deep learning is employed in the study of lncRNAs, thereby providing fresh insights into this fast-growing intersection.

This review is a testament to our commitment to keeping abreast with the most recent advancements in the field, specifically those from 2021 to 2023. As deep learning continues to evolve at a rapid pace, it is imperative to stay up-to-date with the latest research developments. The significance of such a review cannot be overstated, as it will be invaluable to researchers and practitioners who strive to integrate the advancements of deep learning into their lncRNA studies.

## 2. Literature Analysis

### 2.1. Paper Selection Process

The primary aim of the paper selection process was to ensure the inclusion of high-quality, relevant research within the domain. To achieve this, we adopted an algorithmic approach centered around the academic search engine, Web of Science (WOS). Carefully selected search keywords were used, focusing on central themes, such as deep learning, lncRNA, and neural networks, to identify pertinent articles for review.

While recognizing the existence of preprints and conference papers, we chose to concentrate solely on peer-reviewed journal articles. This selection criterion enhances the reliability and validity of the review by ensuring the inclusion of studies that have undergone a stringent review process. This decision was driven by two principal factors. First, the peer-review process serves as a crucial mechanism for maintaining the quality and reliability of scientific literature by subjecting research to thorough scrutiny by domain experts. Second, peer-reviewed journals are traditionally esteemed as trustworthy and credible sources for publishing scientifically sound and influential research.

To maintain the novelty and uniqueness of the review, certain categories of articles, such as review articles and perspective pieces, were intentionally excluded. This approach aimed to emphasize the incorporation of primary research-focused studies, in alignment with the purpose of this review.

The review’s temporal scope was restricted to articles published within the last three years, from 2021 to 2023. This period was selected to assure the relevance and contemporaneity of the review, and to provide a comprehensive understanding of the latest developments and trends in deep learning for lncRNA research. It is worth noting that data collection for 2023 was carried out until May. This was done to ensure that the review’s currency aligns with the most recent advancements in the field. The selected studies are summarized in Table 1.

An exploration of previous deep learning methodologies utilized in the study of lncRNA prior to 2021, as compared to the more recent developments from 2021 to 2023 will certainly provide a comprehensive understanding. A wealth of knowledge on the subject can be found in several representative articles. For example, Baek et al. [102] developed a deep learning-based approach, lncRNAnet, to identify lncRNAs, incorporating recurrent neural networks for RNA sequence modeling and convolutional neural networks for detecting stop codons to obtain an open reading frame indicator. Another study by Fan et al. [103] constructed a powerful predictor, lncRNA–MFDL, to identify lncRNAs by fusing multiple features of the open reading frame, k-mer, the secondary structure, and the most likely coding domain sequence, using deep learning classification algorithms. In 2019, Liu et al. [104] developed a deep learning model, which included a bidirectional long short-term memory model layer and a convolutional layer with three additional hidden layers for distinguishing lncRNAs from mRNAs. These methodologies provided significant advancements in the field. Moreover, the detailed review by Shaath et al. [105] further discusses the interplay between lncRNAs and RBPs and their involvement in epigenetic regulation via histone modifications, highlighting the potential for RNA-based therapeutics in cancer treatment. It is anticipated that the recent surge in research during 2021–2023 has built upon these foundational methodologies and will continue to push the frontier in our understanding and utilization of lncRNA in deep learning.

During the data collection process, we compiled information on the citation count and publication log for each selected article. These details played a significant role in evaluating the scope, impact, and acceptance of the research within the scientific community.

To provide a structured overview of the deep learning methodologies utilized in lncRNA research, the selected papers were categorized, based on the specific objectives of the studies. This categorization contributes to a comprehensive understanding of the deep learning landscape for lncRNA research, by enhancing the understanding of the diverse methodologies employed in the field. The objectives of the studies are summarized in Table 1.

### 2.2. Brief Analysis of Deep Learning Approaches in lncRNA Research

The application of deep learning methodologies to the study of lncRNAs has surged in recent years, as is reflected by the varied research areas illustrated in Table 1. This analysis provides a comprehensive view of the considerable strides made in this emerging field, emphasizing the necessity and relevance of this review.

From the assortment of categories that have emerged, one of the most extensively researched topics is the lncRNA–disease association. Multiple studies have endeavored to harness the predictive capabilities of various deep learning architectures to ascertain the relationship between lncRNAs and diseases [29,34,48]. The prodigious quantity of investigations in this domain highlights the compelling implications these associations could potentially have on clinical diagnostics and therapeutics.

Another critical domain in lncRNA research pertains to lncRNA–protein interactions. In an attempt to uncover the myriad roles that lncRNAs play in molecular biology, numerous researchers have applied deep learning models to predict potential interactions between lncRNAs and proteins [62,70]. By illuminating these interactions, we can elucidate the complex regulatory networks that underpin biological systems.

Similarly, the prediction of lncRNA–miRNA interactions has also gained substantial interest. These investigations capitalize on the ability of deep learning models to discern patterns and predict interactions [72,75], providing vital insights into the modulation of gene expression by lncRNAs.

A further significant facet of lncRNA research that has reaped the benefits of deep learning involves the identification and prediction of characteristics inherent to lncRNAs [88,100]. By leveraging the ability of deep learning models to learn complex representations from data, researchers have made inroads in understanding the fundamental properties that define lncRNAs.

An interesting development has been the application of deep learning in predicting lncRNA subcellular localization. This growing area of study is vital in comprehending the functional mechanisms of lncRNAs since the location of a lncRNA within a cell often indicates its potential role and function.

The investigation of the role lncRNAs play in immune responses using deep learning methods is less explored, yet equally important. While such studies are currently limited in number, they reflect an area that is ripe for further exploration, given the significant implications for immunology and disease pathology. Moreover, identifying lncRNA–protein-coding gene (PCG) associations is another emerging area in lncRNA research where deep learning has been applied.

The diverse application of deep learning in lncRNA research signals an exciting convergence of computational and biological sciences. The rapid development and wide-reaching applicability of deep learning models have significantly enriched our understanding of lncRNAs, highlighting the need to assess and review the state-of-the-art in this field continually. By providing a comprehensive overview of recent studies and emerging trends, this review aspires to be an invaluable resource for researchers exploring the promising intersection of deep learning and lncRNA research.

### 2.3. Distributive Analysis of Publications across Various Journals

In the quest to examine the widespread diffusion of selected articles in a multitude of academic journals, a conspicuous pattern was brought to the fore. The compendium of selected literature exhibits an extensive array of publication outlets, which emphasizes the interdisciplinarity of the topic at hand, i.e., deep learning for lncRNA.

The journal wielding the most considerable influence, as measured by the sheer number of published papers, appears to be *Briefings in Bioinformatics.* This esteemed periodical has been the host to no fewer than 21 of the 75 papers evaluated, thus constituting an impressive 28% of the corpus.

*BMC Bioinformatics* follows on the leaderboard, which accounts for approximately 17% of the selected papers, with a tally of 13. Furthermore, the *IEEE-ACM Transactions on Computational Biology and Bioinformatics* journal has been instrumental in disseminating seven papers within our studied selection.

**Figure 1:**
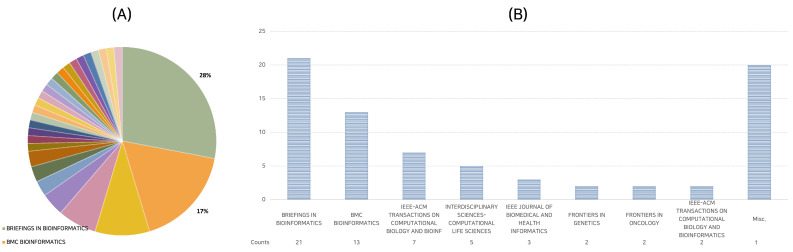
Journal distribution of 75 research papers. (**A**) Proportional representation of papers in various journals, where the distinct journals represented by different colors; (**B**) journals with multiple publications. The Misc. category represents 20 journals with single publications.

This distribution implicitly suggests that specific journals maintain a predilection for publishing research in the domain of deep learning applied to lncRNA. Figure 1 provides a comprehensive analysis, illustrating the allocation of papers among diverse journals.

## 3. Deep Learning Approaches in the Prediction of lncRNA–Disease Associations

LncRNAs have been increasingly recognized as critical components in numerous biological functions and disease processes [106]. The application of deep learning to elucidate lncRNA–disease associations provides an avenue for understanding the complex interactions of lncRNAs with other biological components, including genes and proteins [13]. The richness and complexity of these interactions necessitate computational models that can handle high-dimensional, context-dependent data, a challenge ideally suited to deep learning methodologies.

Notably, trends in lncRNA–disease association research are veering toward the integration of diverse biological data into predictive models. The incorporation of lncRNA sequence data, gene expression data, protein interaction data, and disease phenotype data into deep learning models is enriching the understanding of lncRNA–disease associations [107].

Additionally, the introduction of attention mechanisms has enhanced these models’ ability to focus on the most informative parts of input data. This trend is particularly valuable given the context-specific nature of certain lncRNAs or disease phenotypes. Furthermore, the field is seeing an increased adoption of unsupervised and semi-supervised learning techniques to leverage the abundant amount of unlabeled data in the learning process. The summary of recent studies is provided in Table 2.

### 3.1. Recent Advances from 2021 to 2023

The field of lncRNA–disease associations has seen an influx of diverse prediction models, with the recent trend leaning heavily toward deep learning approaches. The deep learning paradigm, through its effective non-linear data processing capabilities, enables the extraction of complex features and dependencies within the lncRNA–disease association data. The typical models employed in this field incorporate varied architectures, such as deep belief networks (DBNs), convolutional neural networks (CNNs), and attention mechanisms. These methodologies provide improved performance metrics, including the area under the receiver operating characteristic curve (AUC), the area under the precision–recall curve (AUPR), and accuracy values. Notably, graph-based methodologies and multiomics data incorporation are often employed due to their ability to capture complex lncRNA–disease associations and provide comprehensive insights into the role of lncRNA in diseases. However, the choice of the prediction model is largely contingent on the specific requirements of each research, thereby carrying distinct merits and challenges.

In several studies, autoencoders and CNNs were used to improve prediction performance and explore potential disease associations [27,28,38,42,43,44,45,53]. Despite achieving high accuracy rates, a shared disadvantage among these studies is the complexity introduced by the use of autoencoders and CNN. These models require careful parameter tuning and have difficulties integrating information from diverse data types or topologies. Some studies also noted issues with noise in the data, such as [42], which could not effectively remove noisy and irrelevant information.

The second group of studies implemented GNNs to predict lncRNA–disease associations [35,36,38,39,40,41,46,47,50]. These studies demonstrated superior performance in making predictions, and were able to handle multi-view data and efficiently fuse node features, topological structures, and semantic information. However, they have a sensitivity to different datasets and can be complex due to the implementation of multiple attention mechanisms and the integration of multi-layer graph convolutional networks.

The final group of studies utilized matrix factorization or other machine learning techniques, such as support vector machines (SVM) and extreme learning machines (ELM) [33,34,37,49,51,52,54]. They were able to handle complex relationships and diverse features and achieved high accuracy rates. However, they were not without challenges. Some methods required optimal parameter selection, and there were concerns about the quality of the negative samples or graph-based information used in these studies.

In recent studies, while all of these studies have shown promising results in predicting lncRNA–disease associations, each approach has its advantages and potential areas for improvement. Future studies should consider these points when developing prediction models for lncRNA–disease associations.

### 3.2. Emerging Research Trends in Recent Studies

A major shift in recent studies revolves around the application of diverse network architectures, such as DBN, CNN, and attention mechanisms. The primary objective of these trends is to exploit the non-linear data processing potential of deep learning algorithms for complex feature extraction and elucidating the intricate dependencies in lncRNA–disease association data. From a quantitative perspective, models implementing these techniques have persistently demonstrated superior performance metrics.

Another trend gaining momentum is the utilization of graph-based methodologies, with notable contributions by Guo et al. [43], Zeng et al. [33], and Zhao et al. [39]. The inherent strength of these methods lies in their capability to effectively leverage the graph structure of lncRNA–disease associations, thereby capturing the complex interrelationships between lncRNAs and diseases with a higher degree of accuracy. This improved representation of data facilitates more accurate and robust prediction models.

Lastly, the incorporation of multiomics data in lncRNA–disease association prediction, as exemplified by Yuan et al. [45], stands out as a promising trajectory. Quantitatively, this comprehensive approach enables an increased understanding of lncRNA’s role in diseases, substantially enhancing the quality of prediction. While these trends offer numerous advantages, it is important to consider the possible trade-offs. The complexity of these methods can lead to increased computational costs and complexities, and the need for large, high-quality datasets. The benefits and limitations of these trends need to be carefully balanced to maximize their potential in real-world applications.

## 4. Deep Learning Approaches in the Prediction of lncRNA–Protein Interactions

The prediction of lncRNA–protein interactions, a cornerstone in understanding lncRNA functionality, has seen a surge in the use of deep learning models [108]. Predominantly, these models integrate biological features to discern complex patterns. Ensemble learning and hybrid frameworks are often the preferred choices due to their robustness and resilience to overfitting. Moreover, novel methods for quantifying lncRNA gene essentiality are gaining prominence, allowing researchers to further investigate the implications of these interactions in gene functionality. Lastly, the incorporation of cutting-edge techniques, such as serial fusion, capsule networks, and graph autoencoders, signifies the continued evolution of prediction models in lncRNA–protein interaction prediction.

LncRNA–protein interaction (LPI) predictions often start with sequence-based prediction models, which transform the primary sequences of lncRNAs and proteins into feature vectors. Deep learning has shown great potential in predicting lncRNA–protein interactions, with models capable of automatically extracting meaningful features from raw sequence data, including the amino acids of proteins. To manage proteins of different lengths in these models, a process known as zero-padding is utilized, where zeros are added to each sequence up to a common length [109]. This technique is crucial when using raw amino acid sequences as input, as these models require input with the same shape. Furthermore, to tackle the complexity of the three-dimensional structures of proteins, various methods have been proposed, including those that integrate sequence and structure features of the lncRNA and protein, as well as those that apply machine learning algorithms to extract features from sequences [110]. Despite the complexity of plant genome structures, these techniques could be instrumental in predicting lncRNA–protein interactions in plants. A summary of recent studies can be found in Table 3.

### 4.1. Recent Advances from 2021 to 2023

Recent studies can approximately be categorized into three groups: The first cluster comprises methods that apply GNNs, demonstrated in [58] and [63]. They harnessed the power of GNN to predict lncRNA–protein interactions, achieving high AUC and AUPR scores. For instance, BiHo–GNN, a bipartite graph-embedding method based on GNN, reported an impressive AUC of 0.950 and AUPR of 0.899 [58]. iEssLnc, another graph neural network, leveraged meta-path-guided random walks on the lncRNA–protein interaction network to attain an AUC of 0.912 and AUPR of 0.921 [63]. Despite the clear advantages of these methods in terms of accuracy and recall, certain disadvantages exist. For instance, iEssLnc is specialized for essential lncRNA genes and is not generalized for all lncRNA–protein interactions.

In the second group, we see models that have integrated multiple features of lncRNAs and proteins to predict interactions, such as capsule–LPI [59], EnANNDeep [62], LGFC–CNN [64], and LPI–CSFFR [65]. These models demonstrated superior performance by integrating multimodal features or combining raw sequence composition, hand-designed, and structure features. For instance, the capsule–LPI’s multimodal features and multichannel capsule network framework achieved an AUC of 0.951 and AUPR of 0.932, while LGFC–CNN delivered an exceptional AUC of 0.976 and AUPR of 0.970 [59,64]. However, the main limitation of these methods lies in their increased complexity, especially when fusing diverse features. Additionally, some methods, such as capsule–LPI, lack detailed evaluations for each feature [59].

The third group is characterized by models that use deep learning frameworks with different architectures or hybrid approaches. These include DeepLPI [60], DFRPI [61], LPI–deepGBDT [66], LPI–DLDN [67], LPI–HyADBS [68], and RLF–LPI [71]. These models demonstrated high performance due to their unique approaches. For instance, DeepLPI incorporates interactions between lncRNAs and protein isoforms with a hybrid framework of deep neural networks, while LPI–DLDN utilizes a deep learning framework with a dual-net neural architecture. However, these advanced models often require complex integration or feature dimension reduction.

### 4.2. Emerging Research Trends in Recent Studies

The analysis of the recent studies on lncRNA–protein interactions, as summarized in Table 3, reveals several notable trends. Firstly, the integration of deep learning models and various biological features stands out as a common and effective strategy in the field. The studies of Zhou et al. [70], Peng et al. [62,67], and Huang et al. [64] are characteristic of this approach. Their contributions attest to the powerful role deep learning algorithms play in discerning complex patterns, especially when combined with biological features that enhance the models’ understanding of lncRNA–protein interactions.

Secondly, the recent trend of employing ensemble learning and hybrid frameworks is noticeable in studies, such as in the works by Zhou et al. [68] and Song et al. [71]. These studies capitalize on the strength of diverse learning models, making the prediction of lncRNA–protein interactions more robust and less prone to overfitting.

Moreover, the exploration of novel methods for quantifying lncRNA gene essentiality, such as in the study by Zhang et al. [63], further expands the scope of lncRNA research. This signifies a shift toward more comprehensive studies that not only predict interactions but also investigate the implications of these interactions in gene functionality.

Lastly, the efforts of Huang et al. [65], Li et al. [59], and Zhao et al. [69] epitomize the usage of novel techniques, such as serial fusion, capsule networks, and graph autoencoders. These cutting-edge methods contribute to the evolution of prediction models, further advancing the state-of-the-art in lncRNA–protein interaction prediction.

## 5. Deep Learning Approaches in the Prediction of lncRNA–miRNA Interactions

Recent studies on lncRNA–miRNA interactions are primarily centered around the development of deep learning frameworks due to their superior accuracy and cross-species applicability. These interactions, central to post-transcriptional gene regulation, are effectively predicted using sophisticated models such as BoT–Net [72]. The integration of attention mechanisms and neural networks into prediction models is becoming increasingly common, contributing to their improved performance. Hybrid feature mining networks are gaining traction due to their ability to extract useful feature information. Moreover, certain studies are exploring the potential of lncRNA–miRNA interactions in predicting miRNA–disease associations, thereby extending the scope of lncRNA–miRNA research into biomedical applications. However, the model choice largely depends on the specificity and sensitivity requirements of the study, each presenting unique advantages and challenges. A summary of recent studies can be found in Table 4.

### 5.1. Recent Advances from 2021 to 2023

In the domain of predicting lncRNA–miRNA interaction, numerous methodologies have been utilized, each demonstrating varied performance metrics and innovative methodological elements.

Firstly, deep learning models exploiting recurrent neural network (RNN) structures coupled with other strategies have shown substantial performance in predicting lncRNA–miRNA interactions. The authors of [72] utilized a hybrid model consisting of a bidirectional transformer (BoT–Net) and LSTM with DropConnect, in addition to feature pooling. Despite not specifically indicating any disadvantages, the study successfully optimized the lncRNA sequence length, thus improving specificity. Moreover, an optimized ensemble deep learning model, which employs independent RNNs (IndRNNs) and CNNs, was developed by [77]. While the authors have not pointed out any disadvantages, their model’s advantages lie in the improved accuracy achieved through optimal hyperparameter tuning, suitable for large-scale data.

In the second group of studies, graph-based models were significantly utilized, exploiting the potential of deep learning and attention mechanisms. A representative study [73] implemented DWLMI, using the DeepWalk algorithm on an lncRNA–miRNA–disease–protein–drug graph, achieving high accuracy; however, it did not discuss the evaluation of each feature’s influence. Similarly, GCNCRF [74] applied a graph convolutional network (GCN) coupled with a conditional random field (CRF) and an attention mechanism. Despite not mentioning specific disadvantages, the method managed to integrate an lncRNA-miRNA similarity network to achieve high AUC scores. The study by [76] applied a graph neural network-based RNA representation technique, termed ncRNAInter, and demonstrated robust performance and broad applicability across different species.

The third group of studies incorporated hybrid feature mining networks and multi-level information enhancement models to forecast lncRNA–miRNA interaction. PmliHFM [78] uses a hybrid feature mining network tailored for predicting plant miRNA–lncRNA interactions. Despite not specifying any disadvantages, this model uniquely integrated different encodings for miRNA and lncRNA, along with ensemble modules. PmliPEMG [79] employs an ensemble deep learning model, leveraging multi-level information enhancement and a greedy fuzzy decision, thereby incorporating complex fusion features and multi-scale convolutional LSTM networks.

Finally, some studies have developed models that utilize word vector representation and deep feature mining mechanisms. Notably, preMLI [80] is a deep learning model based on rna2vec pre-training and a deep feature mining mechanism. The approach uses rna2vec for RNA word vector representation and displays exceptional cross-species prediction capabilities.

### 5.2. Emerging Research Trends in Recent Studies

As we delve into the intricacies of lncRNA–miRNA associations, one can observe a clear trend toward leveraging advanced machine learning models for predicting these interactions, as evidenced by the studies summarized in Table 4. A noteworthy focus has been on developing deep learning frameworks that offer both improved accuracy and applicability across various species [75,76,77,80].

BoT–Net, a network approach that utilizes long short-term memory networks, provides a good illustration of the potential of these methods [72]. Furthermore, a substantial number of recent studies have shown a strong preference for incorporating attention mechanisms and neural networks into their models, leading to higher performance metrics [74,75].

Moreover, there is an emerging trend of integrating hybrid feature mining networks, which are known to effectively extract useful feature information, thus improving predictive accuracy for lncRNA–miRNA interactions [78]. The commitment to prediction accuracy is further underpinned by the DeepWalk-based method proposed by Yang et al., which offers a high average prediction accuracy [73]. Beyond the application of machine learning in prediction models, some studies have explored the promise of using these interactions to forecast potential miRNA–disease associations, hence pushing the boundaries of lncRNA–miRNA research into biomedical applications.

## 6. Deep Learning Approaches in the Classification and Prediction of lncRNA Characteristics

In the domain of lncRNA characteristics, deep learning has emerged as an effective tool for predicting lncRNA functions, identifying novel lncRNAs, and discovering lncRNA–disease associations. The recent trend is largely geared toward the utilization of machine learning and deep learning models, such as CNN and LSTM, to analyze lncRNA expression profiles, predict lncRNA interactions, and classify different RNA types. Concurrently, the design and application of ensemble methods are growing popular due to their superior predictive performance. Investigations into lncRNA stability and the factors influencing it also provide a more comprehensive understanding of lncRNA biology. However, each approach carries its own strengths and weaknesses, and the choice often depends on the specific objectives of the study.

lncRNAs, in contrast to their protein-coding counterparts, exhibit less conservation, thereby posing considerable challenges for computational models. However, certain unique properties of lncRNAs provide advantageous elements for deep learning. Large lncRNA datasets offer a wealth of data suitable for the training of deep learning algorithms. Additionally, the multi-level regulatory roles of lncRNAs provide multi-modal data (sequence, structure, interactions, expression levels), enabling a comprehensive analysis through multi-modal learning approaches. Importantly, lncRNAs can originate from various genomic regions, including intergenic regions, intronic regions, and antisense transcripts, with each category potentially bearing distinct functional implications. In terms of the prediction of lncRNA characteristics and their source genomic regions, several recent methods have emerged. For instance, multiple studies proposed CNN structures to handle these challenges [82,83,89]. Additionally, LSTM structures were commonly employed for the prediction of lncRNA characteristics [88].

### 6.1. Recent Advances from 2021 to 2023

Deep learning has paved the way for significant advancements in understanding lncRNA characteristics, primarily focusing on the prediction of lncRNA functions and lncRNA identification. Numerous studies have developed models to decipher the hidden complexities of lncRNA biology.

In the domain of predicting lncRNA functions, Zhang et al. [87] utilized an ensemble deep learning model, lncIBTP, for predicting interactions between lncRNAs and different types of biomolecules. The model demonstrated an impressive degree of effectiveness, presenting the potential of deep learning to provide insights into lncRNA functionality. The identification of lncRNAs is another crucial aspect in which deep learning approaches have been employed. Lin and Wichadakul [89] proposed Xlnc1DCNN, a one-dimensional convolutional neural network-based tool. It distinguishes lncRNAs from protein-coding transcripts and provides a rationalization for its predictions. This tool outperformed several others in accuracy and F1-score, demonstrating the effective application of convolutional neural networks in lncRNA identification. Another notable contribution came from Wang et al. [85], who developed LncDLSM, a deep learning-based framework capable of differentiating lncRNAs from protein-coding transcripts without requiring prior biological knowledge. This model excelled in lncRNA identification and exhibited a promising potential for transfer learning.

A unique approach was taken by Zhang et al. [81], who designed a class similarity network for classifying coding and long non-coding RNA. The network explores relationships between input samples and those from the same or different classes, thus obtaining high-level features. The method consistently achieved high accuracy, precision, and F1 scores, signifying its proficiency in lncRNA classification. Ritu et al. [83] also proposed a novel bimodal CNN-based deep learning system, DeepPlnc, which integrated both sequence and structural properties for the identification of plant lncRNAs. DeepPlnc outperformed other tools even when dealing with ambiguous boundaries and incomplete sequences, solidifying its superior applicability in genome and transcriptome annotation tasks.

Promising developments were also made in understanding the stability of lncRNAs. Shi et al. [84] performed a genome-wide RNA-seq study on human lung adenocarcinoma cells and used deep learning-based regression to identify a non-linear relationship between the half-lives of lncRNAs and associated factors. This research illuminated a comprehensive understanding of lncRNA stability, showing the powerful potential of deep learning in elucidating ncRNA characteristics.

Deep learning’s contributions extend to the identification of dual-functional lncRNAs as well; Liu et al. [86] developed LncReader, a deep learning model with a multi-head self-attention mechanism. LncReader outperformed various classical machine learning methods, reiterating the superior performance of deep learning in lncRNA research.

### 6.2. Emerging Research Trends in Recent Studies

In recent research trends, as encapsulated in Table 5, several innovative approaches and methodologies have emerged in the study of lncRNA–disease associations. Various machine learning and deep learning models are increasingly being employed to analyze lncRNA expression profiles, predict lncRNA interactions, and classify different RNA types. These computational tools are crucial in enabling more accurate, efficient, and scalable analyses of lncRNA data.

One remarkable trend observed is the utilization of CNN and LSTM in deep learning models for the prediction and classification of lncRNA [82,83,88,89]. These sophisticated tools offer high accuracy rates and demonstrate robust performance across various datasets. Moreover, they provide an advantage over traditional bioinformatics approaches, which may rely heavily on prior biological knowledge.

Another noteworthy development is the focus on the design and application of ensemble methods, which integrate multiple learning algorithms to obtain better predictive performance [87]. These models, such as WGAN–psoNN and lncIBTP, incorporate advanced concepts, such as NAS for optimal parameter tuning, thus alleviating data imbalance issues.

Furthermore, studies that examine the stability of lncRNAs, and the factors influencing their stability, are gaining traction [84]. These investigations provide a more comprehensive understanding of lncRNA biology and may inform future therapeutic strategies for diseases associated with lncRNA dysfunction.

## 7. Other Deep Learning Research Domains and Utilization of lncRNA-Related Data as Deep Learning Inputs

The exploration of lncRNAs has seen a significant shift toward the use of advanced deep learning models to predict lncRNA subcellular localization and distinguish different lncRNA types. Moreover, studies are increasingly focusing on the role of lncRNAs in immune responses and disease processes, particularly cancer. Novel frameworks, such as deep learning and graph neural networks, are being employed to handle the complex nature of lncRNA sequences and structures. Moreover, deep learning has also found utility in studies investigating the role of lncRNAs in immune responses and disease processes. Researchers are employing sophisticated machine learning techniques to predict lncRNA behavior and correlate it with disease states, offering insights into the complexities of immune responses and disease pathogenesis. A summary of recent studies can be found in Table 6.

### 7.1. Recent Advances from 2021 to 2023

The exploration and prediction of lncRNAs have been widely adopted through advanced computational methods. Among them, DeepLncLoc is a noteworthy tool that uses a subsequence embedding method to retain the order information of lncRNA sequences and incorporates a text convolutional neural network for feature extraction and prediction, proving its effectiveness for lncRNA subcellular localization prediction [90]. Along similar lines, the GM–lncLoc model combines sequence information with graph structure information, leveraging a graph neural network with meta-learning for prediction [92]. This approach demonstrates high accuracy and a promising solution for handling limited sample sizes often seen in lncRNA subcellular localization studies.

Further, GraphLncLoc and PlncRNA–HDeep have shown promising results in lncRNA prediction. The former employs graph convolutional networks and transforms lncRNA sequences into de Bruijn graphs, unveiling sequence patterns and motifs [93]. PlncRNA–HDeep implements a hybrid deep learning model using LSTM and CNN trained on RNA sequences encoded by p-nucleotide and one-hot encodings, achieving high accuracy on the Zea mays dataset and outperforming traditional machine learning methods [94].

Turning to the functional roles of lncRNAs in immune response pathways, the study by [95] utilizes lncRNAs to develop a prognostic model, predicting the survival rates of breast cancer (BC) patients. The model constructs ceRNA networks correlated with the infiltration of CD8 T cells, providing valuable insights into the role of lncRNA in BC. Similarly, the authors of [96] employed a combination of logistic regression and multilayered neural networks to identify lncRNAs related to Bovine Johne’s Disease, revealing potential lncRNA targets in host immunity against Mycobacterium avium infection.

Deep learning applications utilizing lncRNA input data have significantly contributed to the biomedical field. MVMTMDA, a multi-view multitask learning method, predicts miRNA–disease associations from lncRNA–miRNA interactions with an average AUC of 0.8410 ± 0.018 [97]. Another study employed a multimodal deep learning model by integrating histopathological and molecular data to evaluate microsatellite instability in colorectal cancer [98]. This model achieved a high AUC of 0.952 when combining H&E image with DNA methylation data. Moreover, a model built on graph convolution networks with a multichannel attention mechanism predicted miRNA–disease associations based on lncRNA–miRNA interactions, achieving high average AUC values in different cross-validation setups [99].

One particularly innovative approach, WGAN–psoNN, combines the WGAN and particle swarm optimization neural network for predicting lymph node metastasis events using lncRNA expression profiles [100]. Finally, for the identification of lncRNA–PCG associations, the GAE–LGA model utilizes graph autoencoders to integrate multiomics data. This method shows robust capacity in capturing lncRNA–PCG associations, outperforming other machine learning-based approaches [101].

### 7.2. Emerging Research Trends in Recent Studies

The prevailing trend in recent studies, as encapsulated in Table 6, distinctly manifests the increasing significance of computational and deep learning models in the domain of lncRNAs research. A distinct transformation in methodological approaches and predictive models can be observed, mirroring the swift expansion of artificial intelligence and deep learning into biological and medical research fields.

Predominantly, it is seen that many recent studies are oriented toward exploring the subcellular localization of lncRNAs, reflecting the importance of this aspect in understanding the functional roles of lncRNAs. In this quest, researchers are investing significant efforts in constructing robust and sophisticated models that can predict lncRNA localization with high accuracy.

Moreover, the utilization of novel frameworks, such as deep learning and graph neural networks, underscores a consistent theme in these investigations. Such models capitalize on the intricate and complex nature of lncRNA sequences and structures, offering an exciting promise of outperforming traditional machine learning techniques.

Additionally, the studies reflect an increasing interest in the role of lncRNAs in relation to diseases. This manifests in developing models for predicting lncRNA associations with various cell types and disease conditions, in particular, cancer. Such investigations are integral for deepening our understanding of the role of lncRNAs in disease pathogenesis and potential therapeutic interventions.

## 8. Challenges and Future Prospects

LncRNAs have shown substantial promise as novel biomarkers and therapeutic targets in numerous diseases. Notwithstanding, there are still several significant challenges to address and opportunities to seize in the application of deep learning in lncRNA research.

### 8.1. Challenges

Firstly, the scarcity of large, well-annotated datasets is a significant obstacle [111]. Many existing lncRNA-related databases are either relatively small, lack comprehensive annotation, or focus on a particular category of lncRNAs, which restricts the variety and volume of data available for model training and validation. Given the importance of large datasets in deep learning, this poses a substantial challenge to the development of highly accurate and robust models. Moreover, these databases often contain biases, which can inadvertently be learned by the model, leading to biased predictions [112].

Secondly, the heterogeneous nature of lncRNAs themselves presents a significant challenge. LncRNAs are known for their diversity in terms of their biogenesis, structure, function, and localization, which can complicate the feature extraction process in model development [113]. Current methods may not fully capture the complexity and diversity of lncRNAs, leading to the potential loss of valuable predictive information.

Finally, interpretability is a persistent problem in deep learning. Many deep learning models, particularly those with many hidden layers, are often criticized as “black boxes” due to their complex internal computations. This lack of transparency is particularly problematic in biological and medical applications, where understanding the rationale behind predictions is critical [114]. In lncRNA research, this could mean difficulty in identifying key lncRNA features that drive the model’s predictions, impeding the biological understanding of lncRNA functions.

### 8.2. Future Prospects and Directions

Despite these challenges, the application of deep learning in lncRNA research holds significant potential for future advancements. Novel strategies for data augmentation, such as transfer learning, can help to mitigate the issue of data scarcity and improve model performance [115]. Furthermore, the development of more advanced feature extraction techniques that can better capture the complex characteristics of lncRNAs will likely enhance model accuracy and robustness.

Regarding interpretability, recent advancements in model interpretability, such as the development of attention mechanisms and interpretation algorithms, provide promising directions for improving the transparency of deep learning models [116]. In lncRNA research, these tools can aid in identifying important lncRNA features and elucidating their biological significance.

Finally, with the rapid advancement of sequencing technologies and the consequent explosion of genomic data, there is enormous potential for the application of deep learning in lncRNA research. The integration of multiomics data, including transcriptomics, proteomics, and metabolomics, can provide a comprehensive understanding of the complex roles of lncRNAs in biological systems. Developing a multiomics-based deep learning model, especially an advanced multi-attention type encompassing lncRNA, miRNA, mRNA, protein, and their pair matrices, for classification and prediction purposes, would ideally provide a comprehensive understanding of the biological systems. However, there exist substantial challenges in developing such a model, including the requirement for extensive computational resources, the need for expertise in bioinformatics to gather and integrate multiomics data, the difficulty in developing an efficient and effective feature selection strategy due to the high-dimensionality and complexity of the data, and the need to accommodate for the inherent high noise and heterogeneity present in biological data. Despite these challenges, such a direction holds immense promise for future research. For instance, in a recent study [117], mRNA and miRNA data were integrated and used in the analysis. Although lncRNA was not involved in this particular instance, it is anticipated that deep learning methods with multiomics data sources, such as the approach employed in this study, will become a prevalent direction in the future.

## 9. Conclusions

The journey through the landscape of deep learning applications in lncRNA research, as charted in this review, reaches its culmination in this concluding section, where we attempt to knit together the strands of discussions that have threaded their way through the preceding discourse.

An unmistakable trend, revealed through a diligent combination of the literature, is the considerable quantity of investigations that have concentrated on the association between lncRNAs and diseases. This serves as a testament to the recognition within the research community of the profound influence that lncRNAs wield on our physiological and pathological states. These studies have exploited the power of deep learning architectures to decipher the intricate interconnections between lncRNAs and various maladies.

From this multitude of lncRNA–disease association studies, it is clear that the tools and techniques of deep learning are being increasingly harnessed to delve deeper into the complexities of lncRNA functions and their roles in health and disease. This has undoubtedly added significant depth and breadth to our understanding of lncRNA dynamics.

However, it is important to emphasize that this represents only a fraction of the immense potential that deep learning holds for the further exploration of lncRNA. With the accelerating advancements in deep learning methodologies and increasing availability of high-throughput lncRNA data, there is much to look forward to in the realm of lncRNA research.

While the progress achieved thus far in lncRNA research via deep learning is certainly commendable, the journey has just begun. The horizon is replete with possibilities waiting to be uncovered, and it is our hope that this review has inspired further intellectual curiosity and will act as a catalyst for novel studies in this rapidly evolving field.

## Figures and Tables

**Table 1:** Summary of investigations employing deep learning approaches in lncRNA studies between 2021 and 2023.

Research Topics	Deep Learning Approaches
Prediction of lncRNA–disease associations	ACLDA, combining autoencoders, CNN, and attention mechanism [27]; CapsNet–LDA, predicting lncRNA–disease associations using capsule network and attention [28]; DBNLDA, deep belief network-based lncRNA–disease association prediction [29]; Deep learning cluster analysis of lncRNAs in heart failure [30]; DeepMNE, deep multi-network embedding for lncRNA–disease prediction [31]; DHNLDA, deep hierarchical network with stacked autoencoder and ResNet [32]; DMFLDA, deep matrix factorization for predicting lncRNA–disease associations [33]; Dual attention network, enhances the learning of lncRNA–disease feature sets [34]; GCRFLDA, graph convolutional matrix completion-based lncRNA–disease prediction [35]; gGATLDA, lncRNA–disease associations prediction via graph-level attention networks [36]; GSMV, learning of global dependencies and multi-semantics within heterogeneous graphs [37]; GTAN, graph neural network for predicting lncRNA–disease associations [38]; HGATLDA, heterogeneous graph attention network for lncRNA–disease associations [39]; HGNNLDA, heterogeneous graph neural network for lncRNA–disease association [40]; Identifying cancer transcriptome signatures via deep learning interpretation [41]; iLncRNAdis–FB, CNN with fusing biological feature blocks [42]; LDACE, combining extreme learning machine with CNN [43]; LDICDL, identifying lncRNA–disease associations using collaborative deep learning [44]; LGDLDA, predicting disease-related lncRNAs via multiomics data and machine learning [45]; LR–GNN, graph neural network-based prediction of molecular associations [46]; MAGCNSE, lncRNA–disease association prediction via multi-view graph convolutional network [47]; MCA–Net, predicting lncRNA–disease associations using attention CNN [48]; MLMKDNN, predicting ncRNA–disease associations via deep multiple kernel learning [49]; MLGCNET, predicting lncRNA–disease associations using multi-layer graph embedding [50]; Multi-run concrete autoencoder identifying prognostic lncRNAs for cancers [51]; NELDA, predicting lncRNA–disease associations via deep autoencoder models [52]; Novel computational approach, lncRNA–disease prediction via BPSO and ML–ELM [53]; PANDA, graph convolutional autoencoders predicting novel lncRNA–disease associations [54]; Prognostic and diagnostic value of lncRNA in colorectal cancer [55]; VADLP, predicting lncRNA–disease associations with attentional multi-level encoding [56]; VGAELDA, predicting lncRNA–disease associations using variational inference and autoencoders [57].
Prediction of lncRNA–protein interactions	BiHo–GNN, using bipartite graph embedding [58]; Capsule–LPI, a multichannel capsule network for lncRNA–protein interaction prediction [59]; DeepLPI, a multimodal deep learning method for lncRNA–protein isoform interactions [60]; DFRPI, deep autoencoder and marginal Fisher analysis [61]; EnANNDeep, ensemble-based framework with adaptive k-nearest neighbor for the lncRNA–protein interaction [62]; iEssLnc, graph neural network-based estimation of lncRNA gene essentiality [63]; LGFC–CNN, using deep learning with feature combination [64]; LPI–CSFFR, CNN-based lncRNA–protein interaction prediction with serial fusion and feature reuse [65]; LPI–deepGBDT, gradient boosting decision trees-based lncRNA–protein interaction identification [66]; LPI–DLDN, dual-net neural architecture for lncRNA–protein interactions prediction [67]; LPI–HyADBS, hybrid framework with DNN, XGBoost, SVM for lncRNA–protein interaction [68]; LPICGAE, predicting lncRNA–protein interactions using combined graph autoencoders [69]; PRPI–SC, ensemble deep learning for plant lncRNA–protein interactions prediction [70]; RLF–LPI, ensemble learning framework with residual LSTM and fusion attention [71].
Prediction of lncRNA–miRNA interactions	BoT–Net, efficient lncRNA–miRNA interaction prediction using the bag of tricks-based neural network [72]; DeepWalk–LMI, inferring lncRNA–miRNA associations via comprehensive graph [73]; GCNCRF, predicting lncRNA–miRNA interactions using graph convolution and conditional random field [74]; MD–MLI, predicting lncRNA–miRNA interactions using multiple features and hierarchical deep learning [75]; ncRNAInter, a novel strategy using a graph neural network to discover lncRNA–miRNA interactions [76]; Optimized ensemble deep learning, predicting plant lncRNA–miRNA based on artificial gorilla troops algorithm [77]; PmliHFM, plant lncRNA–miRNA interaction prediction via hybrid feature mining network [78]; PmliPEMG, multi-level information enhancement and greedy fuzzy decision for plant lncRNA–miRNA interaction prediction [79]; preMLI, uncovering potential lncRNA–miRNA interactions through pre-training and deep feature mining [80].
Classification and Prediction of lncRNA characteristics	Class similarity network, identifying lncRNAs using relationships among samples [81]; DeepLncPro, CNN for identifying lncRNA promoters [82]; DeepPlnc, high accuracy plant lncRNA identification using bimodal CNN [83]; Genome-wide analysis, exploring features related to human lncRNA stability [84]; LncDLSM, lncRNA identification using the deep learning-based sequence model [85]; LncReader, identifying dual-functional lncRNAs using multi-head self-attention [86]; lncIBTP, predicting interaction biomolecule type for a given lncRNA using ensemble deep learning [87]; RNA prediction based on neural network integration of CNN and Bi-LSTM [88]; Xlnc1DCNN, interpretable deep learning model, lncRNA identification using 1D CNN [89].
Prediction of lncRNA subcellular localization	DeepLncLoc, a deep learning framework for lncRNA subcellular localization using subsequence embedding [90]; EVlncRNA–Dpred, an improved prediction method of experimentally validated lncRNAs using deep learning [91]; GM–lncLoc, lncRNA subcellular localization prediction based on graph neural network with meta-learning [92]; GraphLncLoc, predicting lncRNA subcellular localization using graph convolutional networks and sequence-to-graph transformation [93]; PlncRNA–HDeep, a plant long non-coding RNA prediction method that utilizes hybrid deep learning with two encoding styles [94].
Prediction of functional roles of lncRNAs in immune response pathways	CD8–Net, constructing ceRNA networks for CD8 T cells in breast cancer [95]; JD–lncRNA–ID, identifying lncRNA associated with bovine Johne’s disease using neural networks and logistic regression [96];
Deep learning applications through the utilization of lncRNA input data	MVMTMDA, predicting miRNA–disease associations through lncRNA–miRNA interactions [97]; Predicting microsatellite instability in colorectal cancer using multimodal deep learning [98]; Predicting miRNA–disease associations, a method based on lncRNA–miRNA interactions and graph convolution networks [99]; WGAN–psoNN, tumor lymph node metastasis prediction using WGAN and psoNN [100].
Identification of lncRNA–protein-coding gene (PCG) associations	GAE-LGA, deep learning prediction of lncRNA–PCG associations with cross-omics correlation learning [101].

**Table 2:** Summary of recent studies regarding the prediction of lncRNA–disease association. It is important to acknowledge that each study utilized diverse datasets, cross-validation methods, and simulation settings to assess accuracy, thus rendering direct comparisons potentially inconclusive. The best accuracy was selected if the model was assessed with various datasets.

Ref.	Methods	Accuracy	Merits	Disadvantages
[27]	ACLDA, fully connected autoencoder and CNN with attention mechanisms	AUC: 0.956 AUPR: 0.393	Improved prediction performance; potential for disease exploration	Failure to deeply integrate topology information
[28]	CapsNet–LDA, attention mechanism, stacked autoencoder, adaptive allocation, CapsNet architecture	AUC: ≈0.97	Superior performance and robustness, good generalization	Complexity due to the use of vector neurons
[29]	DBNLDA, node embedding, DBN, and neural network regression model	AUC: 0.96 AUPR: 0.968	Better prediction performance, potential in disease therapy	Complexity due to diverse network structured data
[30]	Deep belief network for HF lncRNAs, topic model-based network cluster analysis	AUC: 0.92	Identification of key lncRNAs, potential diagnostic biomarkers	Focused only on HF, needs wider disease scope
[31]	DeepMNE, deep multi-network embedding, network fusion based on deep learning	AUC: 0.9462 AUPR: 0.9505	Superior performance in identifying new associations between lncRNAs and diseases	Complexity due to multiomics data integration
[32]	DHNLDA, deep hierarchical network, stacked autoencoder, ResNet, stacked ensemble module	AUC: 0.975	High predictive performance, potential for identifying disease associations	The complexity of the hierarchical network structure
[33]	DMFLDA, deep matrix factorization, non-linear hidden layers	AUC: 0.8393	Better than SIMCLDA, TPGLDA, MFLDA, LDAP; capable of complex relationship representation	More experimentation needed
[34]	Dual attention network method, feature fusion networks	AUC: 0.914 AUPR: 0.339	Superior performance in recognizing potential lncRNA-disease associations across 10 categories of diseases	Not specified
[35]	GCRFLDA, scoring lncRNA–disease associations, graph convolution matrix completion, conditional random field	AUC: 0.9630	Outperforms DMFLDA and LDASR; confirmed associations in case studies	Not specified
[36]	gGATLDA, GNN model with enclosing subgraphs and integrated feature vectors	AUC: 0.9888 AUPR: 0.9890	High accuracy, F1-Score, stable prediction performance	Sensitivity to different datasets, possible lower accuracy and precision for some data
[37]	GSMV, global dependencies, semantic information, multi-view features, self-attention mechanism, dilated convolution-based learning module	AUC: 0.983 AUPR: 0.589	Superior performance, rich semantic information extraction	Not specified
[38]	GTAN, GNN with three attention mechanisms, multi-layer CNN	AUC: 0.983 AUPR: 0.454	High accuracy; potential for discovering new disease-related lncRNA candidates	Not specified
[39]	HGATLDA, heterogeneous graph attention network, meta-paths	AUC: 0.9424 AUPR: 0.4701	Efficient in fusing node features, topological structures and semantic info; handles imbalance in prediction	Not specified
[40]	HGNNLDA, GNN, heterogeneous network, restart random walk, type-based neighbor aggregation	AUC: 0.9786 AUPR: 0.8891	Exploits network topology for better predictions; potential for predicting new diseases	Not specified
[41]	Feedforward neural networks based on gene expression	Acc.: 0.9862 AUPR: 0.9988	High prediction accuracy, useful for identifying commonly deregulated features across cancer types	Model performance can vary with fewer samples
[42]	iLncRNAdis–FB, fusing biological feature blocks using CNN	AUC: 0.909 AUPR: 0.363	Better performance compared to other predictors; web server for potentially associated disease detection	Fails to remove noise and irrelevant information
[43]	LDACE, CNN for feature mining, ELM for prediction tasks	Acc: 0.8252 AUC: 0.8995	Remarkable performance in cross-validation, case studies for robustness	Adequate feasibility in bioinformatics not verified
[44]	LDICDL, collaborative deep learning, autoencoder for feature denoising, matrix decomposition for potential association prediction	AUC: 0.8651 AUPR: 0.0306	Outperforms other methods, handles new lncRNA or diseases	Limited matrix decomposition for prediction
[45]	LGDLDA, non-linear feature learning of neural networks and node representation approximation	AUC: 0.926	Better stability and performance in cancer-related lncRNA prediction, utilized diverse data	Complexity due to diverse data integration
[46]	LR–GNN, GNN based on link representation for predicting molecular associations; GCN encoder for node embedding and layer-wise fusing rule for the output	AUC: 0.9474 AUPR: 0.9497	Outperforms state-of-the-art methods in molecular association predictions, versatile in different association types	May need an optimal layer-fusing rule design for performance
[47]	MAGCNSE, multi-view attention graph convolutional network and stacking ensemble model	Acc.: 0.9395	Enhanced performance in lncRNA–disease associations predictions, effective utilization of multi-view data	Complexity of the stacking ensemble model
[48]	MCA–Net, multi-feature coding, six similarity features, attention convolutional neural network	Acc.: 0.967 AUC: 0.994	Outperforms state-of-the-art methods on three datasets	Requires careful tuning of model parameters
[49]	MLMKDNN, multi-layer multi-kernel DNN, feature matrices, kernel space mapping, DNN	AUC: 0.976 AUPR: 0.92	High AUPR value on three types of datasets	Complexity due to multiple-feature integration
[50]	MLGCNET, graph convolutional network, reconstructed similarity networks, latent feature representations of nodes, extra trees method	AUC: 0.982 AUPR: 0.408	Superior prediction performance, effective for specific diseases	Complexity due to multi-layer graph convolutional network
[51]	mrCAE, multi-run concrete autoencoder, lncRNA expression profiles, multiple runs	Acc.: 0.95	Better feature selection, identified 128 lncRNAs related to 12 cancers	Stochastic nature of CAE may affect reproducibility
[52]	NELDA, SVM classifier-based model, deep autoencoder models, weighted average strategy	AUC: 0.9827	Superior AUC result, high potential in disease diagnosis and treatment	Relies on quality negative samples selection
[53]	Novel method using BPSO and ELM models, wrapper feature extraction method	Acc.: ≈0.93	Highest accuracy, effective in predicting key lncRNA–disease relationships	Necessity of optimal lncRNA subset selection
[54]	PANDA, graph-based method, heterogeneous graph, graph autoencoder, neural network for prediction	AUC: 0.976 AUPR: 0.956	Impressive AUC-ROC, promising for predicting novel lncRNA–disease associations	Depends on the quality of graph-based information
[55]	Prognostic and diagnostic value of lncRNA in colorectal cancer with the classification of mRNA, lncRNA, and circRNA in exosomes	ND	Potential for exploring immune infiltration levels in CRC, diagnosis, therapy, and prognosis	No quantitative classification performance evaluation
[56]	VADLP, deeply embedded node attributes, weighted inter-layer and intra-layer edges, convolutional autoencoder, variance autoencoder	AUC: 0.956 AUPR: 0.449	Improved recall rates, powerful in discovering true disease-related lncRNAs	Complex due to multiple representations
[57]	VGAELDA, integrates variational inference and graph autoencoders, alternate training via variational inference	AUC: 0.968 AUPR: 0.838	Robustness and preciseness for predicting unknown lncRNA–disease associations	Complex due to the alternate training approach

**Table 3:** Summary of recent studies regarding the prediction of lncRNA–protein interaction. It is important to acknowledge that each study utilized diverse datasets, cross-validation methods, and simulation settings to assess accuracy, thus rendering direct comparisons potentially inconclusive. The best accuracy was selected if the model was assessed with various datasets.

Ref.	Methods	Accuracy	Merits	Disadvantages
[58]	BiHo–GNN, bipartite graph embedding based on GNN	AUC: 0.950 AUPR: 0.899	High AUC and recall, outperforms existing methods	Not specified
[59]	Capsule–LPI, multimodal features, multichannel capsule network framework	AUC: 0.951 AUPR: 0.932	Superior performance, integration of multimodal features	Absence of detailed evaluation for each feature
[60]	DeepLPI, interactions between lncRNAs and protein isoforms with the hybrid framework of deep neural networks	AUC: 0.866 AUPR: 0.703	Use of isoforms, application of multiple instance learning	Lower performance metrics compared to other methods
[61]	DFRPI, deep autoencoder and marginal Fisher analysis, random forest-based predictor	AUC: 0.906	Constructing a discriminative feature space, high precision	Necessity to generate a reasonable and effective feature space
[62]	EnANNDeep, an ensemble-based framework with an adaptive k-nearest neighbor classifier and deep models	AUC: 0.916 AUPR: 0.905	Incorporates multiple source features, performs well in cross-validations	May produce prediction bias with single dataset evaluation
[63]	iEssLnc, graph neural network with meta-path-guided random walks on the lncRNA–protein interaction network	AUC: 0.912 AUPR: 0.921	Provides quantitative essentiality scores for lncRNA genes	Specific to essential lncRNA genes, not general lncRNA–protein interactions
[64]	LGFC–CNN, deep learning-based prediction combining raw sequence composition, hand-designed, and structure features	AUC: 0.976 AUPR: 0.970	Multiple-feature integration, highly accurate performance	Not specified
[65]	LPI–CSFFR, a feature fusion method based on CNN with feature reuse and serial fusion	AUC: 0.879	Integrates diverse features of lncRNAs and proteins, high accuracy	Requires complex feature fusion
[66]	LPI–deepGBDT, multiple-layer deep framework based on gradient boosting decision trees	AUC: 0.9073 AUPR: 0.8849	Uses diverse biological information of lncRNAs and proteins	Limited application for new lncRNAs or proteins
[67]	Deep learning framework with dual-net neural architecture, LPI–DLDN	AUC: 0.911 AUPR: 0.898	Best average AUC and AUPR, outperforms six other LPI prediction methods	Requires dimension reduction for feature concatenation
[68]	LPI–HyADBS, AdaBoost-based feature selection, combined with DNN, XGBoost, C-SVM	AUC: 0.851 AUPR: 0.841	Hybrid approach integrates multiple classifiers, surpasses six other models	Requires complex integration of classifiers
[69]	LPICGAE, combined graph autoencoders	AUC: 0.974 Acc.: 0.985	Low-dimensional representations, outperforms six other computational methods	May need alternate loss minimization for optimal results
[70]	PRPI–SC, ensemble deep learning model using stacked denoising autoencoder and CNN	Acc.: 0.889 AUC: 0.950	Predicts plant LPIs, generalizes well beyond plant data	Only reports accuracy for plant data
[71]	RLF–LPI, AE–ResLSTM with fuzzy decision	Acc.: 0.921 AUC: 0.980	Potential for high performance due to the use of AE–ResLSTM and fuzzy decision	Not specified

**Table 4:** Summary of recent studies regarding the prediction of the lncRNA–miRNA interaction. It is important to acknowledge that each study utilized diverse datasets, cross-validation methods, and simulation settings to assess accuracy, thus rendering direct comparisons potentially inconclusive. The best accuracy was selected if the model was assessed with various datasets.

Ref.	Methods	Accuracy	Merits	Disadvantages
[72]	BoT–Net, LSTM with DropConnect, feature pooling	Acc.: 0.8738 AUC: 0.9449	Optimized lncRNA sequence length, improved specificity	Not specified
[73]	DWLMI, DeepWalk on lncRNA–miRNA–disease–protein–drug graph	Acc.: 0.9522 AUC: 0.9856	High accuracy, incorporation of multi-dimensional data	Evaluation of each feature’s influence not discussed
[74]	GCNCRF, GCN with Conditional random field and attention mechanism	AUC: 0.947	High AUC, inclusion of lncRNA/miRNA similarity network	Not specified
[75]	MD–MLI, hierarchical deep learning with multiple features	Acc.: 0.9859	High accuracy, uses multiple sequence-derived features	Not specified
[76]	ncRNAInter, graph neural network-based RNA representation	AUC: 0.973 AUPR: 0.975	Robust performance, universal applicability for diverse species	Not specified
[77]	Optimized ensemble deep learning model, leverages independent recurrent neural networks and convolutional neural networks	Acc.: 0.977	Improved accuracy via optimal hyperparameter tuning, works with large-scale data	Not specified
[78]	PmliHFM, hybrid feature mining network for predicting plant miRNA–lncRNA interactions	Acc.: 0.938 AUC: 0.963	Different encodings for miRNA and lncRNA, ensemble module integration	Not specified
[79]	PmliPEMG, ensemble deep learning model with multi-level information enhancement and greedy fuzzy decision	Acc.: 0.888 AUC: 0.971	Incorporates the fusion of complex features and multi-scale convolutional long short-term memory networks	Not specified
[80]	preMLI, deep learning model based on rna2vec pre-training and deep feature mining mechanism	Acc.: 0.924 AUC: 0.977	Uses rna2vec for RNA word vector representation, excellent cross-species prediction capabilities	Not specified

**Table 5:** Summary of recent studies regarding the classification and prediction of lncRNA characteristics. It is important to acknowledge that each study utilized diverse datasets, cross-validation methods, and simulation settings to assess accuracy, thus rendering direct comparisons potentially inconclusive.

Ref.	Methods	Accuracy	Merits	Disadvantages
[81]	Class similarity network, Siamese neural network-inspired model	Acc: 0.9843	Directly explores relationships among input samples, achieving high-level features	Insufficient exploration of relationship among samples
[82]	DeepLncPro, convolutional neural network model for identifying lncRNA promoters	Acc: 0.8622	Superior to existing methods, can extract and analyze transcription factor binding motifs	Not specified
[83]	DeepPlnc, bimodal CNN-based system for plant lncRNA discovery	Acc: 0.9806 AUC: 0.9955	High accuracy, can handle ambiguous boundaries and incomplete sequences	Not specified
[84]	Deep learning-based regression for genome-wide analysis of lncRNA stability	ND (Mainly focused on genome-wide analysis)	Comprehensive understanding of lncRNA stability	Absence of a detailed quantitative prediction model for half-lives
[85]	LncDLSM, deep learning-based sequence model for lncRNA identification	Acc: 0.9652 AUC: 0.9962	No dependency on prior biological knowledge, can be applied to other species	Not specified
[86]	LncReader, multi-head self-attention mechanism	Acc.: 0.969 AUC: 0.803 AUPR: 0.265	Excels in dual-functional lncRNA identification; superior performance compared to classical machine learning methods	Not specified
[87]	lncIBTP, ensemble deep learning approach	Acc.: 0.704 AUC: 0.790 AUPR: 0.642	Novel approach to predicting interaction biomolecule type for lncRNA, performs well on 5-fold cross-validation	Does not specifically predict lncRNA functions
[88]	CNN and Bi–LSTM combined model for RNA prediction	Acc.: 0.977	Superior classification effect compared to a single model, demonstrates strong generalization capacity	Not specified
[89]	Xlnc1DCNN, 1D convolutional neural network	Acc.: 0.945 AUC: 0.983	Outperforms other tools in accuracy and the F1-score on the human test set, provides prediction explanations	Inconsistent annotations among public databases

**Table 6:** Summary of other recent studies and utilizations of lncRNA-related data as deep learning inputs.

Ref.	Methods	Merits	Disadvantages
[90]	DeepLncLoc: Uses a subsequence embedding method that keeps the order information of lncRNA sequences. Utilizes a text convolutional neural network for feature extraction and prediction.	Effective for lncRNA subcellular localization prediction. Preserves sequence order information.	Depends on the quality of subsequence embedding. Might miss some complex patterns.
[91]	EVlncRNA–Dpred: Uses deep learning algorithms to distinguish experimentally validated lncRNAs from mRNAs and high-throughput lncRNAs. Utilizes a three-layer deep learning neural network with a small convolutional neural network.	Provides a method for prioritizing potentially functional lncRNAs for experimental validations.	Accuracy can be limited by the small dataset sizes.
[92]	GM–lncLoc: Uses a graph neural network with meta-learning to predict lncRNA subcellular localization. Combines the initial sequence information with graph structure information to extract features.	Shows high accuracy, holds the potential to solve the problem of limited samples in lncRNA subcellular localization.	Performance heavily depends on the quality of the graph’s structure information.
[93]	GraphLncLoc: Uses graph convolutional networks to predict lncRNA subcellular localization. Transforms lncRNA sequences into de Bruijn graphs.	Can reveal sequence patterns and motifs. Demonstrates robustness against k-mer frequency features.	Transforming sequences into graphs might lead to the loss of certain information.
[94]	PlncRNA–HDeep: Hybrid deep learning model that uses LSTM and CNN trained on RNA sequences encoded by p-nucleotide and one-hot encodings.	Achieves high accuracy on the Zea mays dataset. More effective than traditional machine learning methods and some existing tools.	Model complexity could lead to overfitting.
[95]	Uses lncRNAs to develop a prognostic model that predicts the survival rates of BC patients. Constructs ceRNA networks correlated with the infiltration of CD8 T cells.	Can help understand the role of lncRNA in BC. Useful for predicting patient prognosis.	Relies on the bioinformatic prediction of CD8 T cell abundances, which might not always be accurate.
[96]	A combined approach using logistic regression and multilayered neural networks to identify lncRNAs related to Bovine Johne’s Disease.	Identifies potential lncRNA targets in host immunity against Mycobacterium avium infection.	Not specified
[97]	Multi-view multitask learning method that predicts microRNA–disease associations from lncRNA–microRNA interactions.	Developed the MVMTMDA model for predicting miRNA–disease associations, achieving an average AUC of 0.8410 ± 0.018.	Requires comprehensive lncRNA–miRNA interaction data.
[98]	Multimodal deep learning integrating histopathological and molecular data to evaluate the microsatellite instability of colorectal cancer.	The developed model achieves a high AUC of 0.952 when combining an H&E image with DNA methylation data.	Accuracy decreases when combining an H&E image with all types of molecular data.
[99]	Uses graph convolution networks with multichannel attention mechanism to predict miRNA–disease associations based on lncRNA–miRNA interactions.	Achieves average AUC values of 0.8994, 0.9032, and 0.9044 in different cross-validation setups.	Lacks comparative analysis with non-deep learning models.
[100]	WGAN–psoNN: Combines the Wasserstein distance-based generative adversarial network (WGAN) and particle swarm optimization neural network (psoNN) to predict lymph node metastasis events using lncRNA expression profiles.	Reduces the requirement for deep learning data quantity and architecture selection.	Uses the novel neural network architecture search (NAS) method, which is untested in other studies.
[101]	GAE–LGA: Uses graph autoencoders to integrate multiomics data and identify lncRNA–PCG associations.	Shows strong robustness in capturing lncRNA–PCG associations and outperforms other machine learning-based methods.	Not specified

## Data Availability

No new data were created or analyzed in this study.
